# A Comprehensive Probabilistic Risk Assessment Strategy of Aflatoxin B1 Exposure from Medical Areca Nuts Consumption

**DOI:** 10.3390/toxins17050252

**Published:** 2025-05-19

**Authors:** Tian-Tian Zuo, Ya-Zhong Zhang, Hong-Yan Zhai, Rong-Yao Chen, Jing-Zhe Pu, Hong-Yu Jin, Jing Liu, Xian-Long Cheng, Feng Wei

**Affiliations:** 1 National Key Laboratory of Medicine Regulatory Science, National Institutes for Food and Drug Control, Beijing 100050, China; 2 Anhui Institutes for Food and Drug Control, Hefei 230051, China

**Keywords:** aflatoxin B_1_, probabilistic risk, Monte Carlo simulation, MOE, liver cancer, medical areca nuts

## Abstract

Aflatoxin B_1_ (AFB_1_) is widely found and substantially impends public health. The current work aimed to assess AFB_1_ in medical areca nuts in China. The average content of AFB_1_ was 13.0 μg/kg, and the maximum content was 146.0 μg/kg. Furthermore, a comprehensive probabilistic risk assessment approach considering combined utilization of a Monte Carlo simulation with the margin of exposure (MOE) and quantitative liver cancer risk (HCC) strategies was developed for assessing the human health risk of AFB_1_ from consuming medical areca nuts for the first time. The MOE values of AFB_1_ in samples for no more than the 75th percentile were less than the threshold of 10,000 for both men and women. The estimated 90th percentile to the maximum of the HCC values for males and the estimated 75th percentile to the maximum of the HCC values for females were higher than one in a million upon exposure to medical areca nuts, indicating an unacceptable liver cancer risk. Sensitivity analysis demonstrated that for both MOE and HCC approaches, AFB_1_ content was the parameter with the greatest effects on the results, followed by the exposure frequency (EF) and daily intake rate (IR). This study is the first of this kind, demonstrating the applicability of stochastic exposure evaluation techniques for the precise and scientific assessment of the health risk of AFB_1_ in medical areca nuts, with the main purpose of minimizing human cancer risk.

## 1. Introduction

Mycotoxin, a word derived from Greek (‘mykes’) and Latin (‘toxicum’) terms, is a toxic low-molecular-weight secondary metabolite produced by certain fungi under suitable environmental conditions. Mycotoxins are responsible for severe crop diseases and endanger human health, particularly regarding carcinogenic effects; moreover, they may damage internal organs, cause disorders of the reproductive system, suppress immune function, and induce genotoxicity in humans [1,2,3,4,5,6,7]. There are more than 300 discovered mycotoxins, including aflatoxin, ochratoxin, fumonisin, vomitoxin, patulin, and T-2 mycotoxin as the common ones [8].

Aflatoxins are the most widespread mycotoxins. They represent secondary metabolites produced by *Aspergillus flavus*, *Aspergillus parasiticus*, *Aspergillus nomius*, and *Aspergillus tamarii* at high temperatures and humidity [9]. Among the four main aflatoxins (AFs), i.e., AFB_1_, AFB_2_, AFG_1_, and AFG_2_, AFB_1_ constitutes the commonest and most toxic type in nature and is considered, by the International Agency for Research on Cancer (IARC), to be a group one carcinogen [10,11]. AFB_1_’s acute toxicity is 10- and 68-fold higher than the levels reported for potassium cyanide and As_2_O_3_, respectively. This toxin causes serious damage to human and animal organs, including abdominal pain, diarrhea, vomiting, anorexia, lung edema, depression, and fatty liver [12]. Moreover, exposure to AFB_1_ induces immune function impairment and many malignancies, especially liver cancer [13,14,15,16].

The World Health Organization (WHO), as well as various countries and regions, have implemented stringent regulations on aflatoxin residues in food and agricultural products to minimize AFB_1_ exposure in humans. The Codex Alimentarius Commission, Joint FAO/WHO Food Standards Program, adopted a maximum level of 15 µg/kg for total aflatoxins for unprocessed peanuts and tree nuts and 10 µg/kg for ready-to-eat tree nuts [17]. In China, the limit of 20 µg/kg was set by the National Food Safety Standard for AFB_1_ in peanuts and maize [18]. In the European Commission regulation (EC) No. 1881/2006, the maximum limit of AFB_1_ for peanuts and cereals is 2 ng/g [19]. The United States imposed a maximum level for AFB_1_ for corn- and peanut-derived products of 20 ng/g [20].

Food and feed products can be contaminated by aflatoxins in various ways, and aflatoxins can directly or indirectly enter the human food chain and threaten human health and life [21]. In addition, traditional Chinese medicine (TCM) preparations can also be susceptible to moisture, which is favorable for aflatoxin formation, during collection, storage, processing, and transportation, which leads to aflatoxin contamination [22]. In particular, herbal medicines such as seeds, fruits, and fermented products are especially susceptible to aflatoxin contamination. As the dried mature seed of *Areca catechu* L., the medical areca nut is widely distributed in South and Southeast Asia, with a long history of consumption in China, India, Indonesia, Myanmar, Malaysia, and Bangladesh [23,24,25,26]. The main compounds of the medical areca nut encompass polysaccharides, polyphenols, alkaloids, and fatty acids [27,28]. The pharmacological and biological effects of medical areca nuts include anthelmintic, stimulant, gastrointestinal motility-promoting, antioxidant, anti-aging, anti-depressant, anti-inflammatory, and anti-tumor activities [29,30,31,32,33,34,35]. Therefore, medical areca nuts are usually used to treat multiple diseases such as malaria, depression, hypertension, hyperlipidemia, and diabetes.

The medical areca nuts are easily contaminated by AFB_1_. The Food and Agriculture Organization of the United Nations and WHO (FAO/WHO) have collaborated for a long time to examine the safety of AFB_1_ in food products. The risk assessment, the major scientific part of risk analysis, was mainly designed to help make proper decisions for protecting health [36,37]. Owing to the strong genotoxicity and carcinogenicity of AFB_1_, there are no health guidance parameters, involving daily tolerable intake (TDI), temporary daily tolerable intake (PTDI), and temporary weekly tolerable intake (PTWI), established by international organizations. The previous approach suggested for exposure assessment is to apply the as low as reasonably achievable (ALARA) principle [38]. For example, the Joint Expert Committee on Food Additives (JECFA) carried out the first risk assessment of AFB_1_ in 1987 [38]. Due to insufficient toxicological research data on aflatoxins, applying the ALARA principle was suggested. The levels of AFB_1_ should be kept as low as possible to reduce health risks. With the continuous development and improvement of exposure assessment research on AFB_1_, risk assessment methods for AFB_1_ have gradually shifted from simple qualitative methods to semi-quantitative and quantitative techniques [39].

In recent studies, the margin of exposure (MOE), based on the ratio of a toxicological threshold from animals (e.g., benchmark dose limit) is the most commonly used way for characterizing carcinogenic and genotoxic risks for mycotoxins in food [40]. To the best of our knowledge, little data regarding health risk assessment approaches applicable to AFB_1_ in TCMs are available. With the ever-lasting use of medical areca nuts, especially in Asia-Pacific regions, and the increasing use of TCM worldwide, the safety of this type of TCM is considered an important issue for the public. AFB_1_ contamination in the medical areca nut is of great concern, affecting the quality safety of medical areca nuts. Moreover, medical areca nuts used as TCM constituents differ from food, being employed for treating diseases and less often consumed than food. In order to protect public health, it is imperative to develop scientific tools for the risk assessment of AFB_1_ in medical areca nuts. Therefore, this study aimed (1) to investigate AFB_1_ occurrence in medical areca nuts, (2) to develop the output of exposure estimates of AFB_1_ in medical areca nuts based on a real-time scenario, and (3) to establish the first probabilistic risk of dietary exposure to AFB_1_ in medical areca nuts by both the margin of exposure (MOE) approach and liver cancer risk estimation.

## 2. Results and Discussion

### 2.1. Results of AFB_1_ Prevalence in Medical Areca Nuts

The detection rate of AFB_1_ in all samples was 88.0%. The maximum permissible limit in the 2020 edition of Chinese Pharmacopoeia for AFB_1_ in herbal medicines is 5 μg/kg. According to the maximum permissible limit for herbal medicines in the Chinese Pharmacopoeia, the qualified rate for AFB_1_ in 158 medical areca nut samples was 62.0% (qualified rate = $\frac{\mathrm{number}\mathrm{of}\mathrm{samples}\mathrm{whose}\mathrm{affatoxinl}\mathrm{contents}\mathrm{were}\mathrm{lower}\mathrm{than}\mathrm{the}\mathrm{maximum}\mathrm{permissible}\mathrm{limits}}{\mathrm{total}\mathrm{number}\mathrm{of}\mathrm{samples}}\times 100\%$). Generally, the P_25_, average, P_50_, and P_75_ levels of AFB_1_ in all medical areca nut samples were 1.0, 13.0, 3.0, and 18.0 μg/kg, respectively (Table 1). The maximum level of AFB_1_ in medical areca nut samples was 146.0 μg/kg, which was up to 28 times higher than the allowable limit. Among these samples, the detection rates of AFB_1_ in roasted and stir-fried medical areca nuts were 0 and 62.5%, respectively. The average, P_50_, and P_75_ levels of AFB_1_ in stir-fried medical areca nut samples were 7.7, 1.5, and 12.5 μg/kg, respectively. The maximum level of AFB_1_ in stir-fried medical areca nut was 31.0 μg/kg, which was up to five times higher than the limit. AFB_1_ was not detected in roasted medical areca nut samples using the same method for medical areca nuts and stir-fried medical areca nuts.

### 2.2. Risk Assessment Results

#### 2.2.1. Estimated Daily Intake of AFB_1_ for Medical Areca Nuts

The point estimation method for risk assessment usually yields uncertain outcomes. To overcome this shortcoming, the probability distributions of crucial exposure indexes were applied in the present study. The evaluation of the probability distributions of IR and EF were obtained from questionnaire data for achieving a highly realistic probabilistic model suitable for medical areca nuts. The parameter values and optimal probability distributions of these parameters are presented in Table 2.

**Figure 1:**
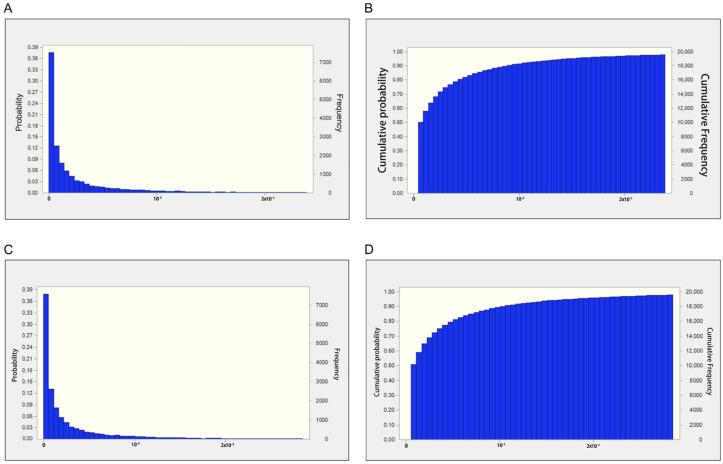
(**A**) Probabilistic estimation of EDI (ng/kg/d) of investigated AFB_1_ for males. (**B**) Cumulative probabilistic estimation of EDI (ng/kg/d) of investigated AFB_1_ for males. (**C**) Probabilistic estimation of EDI (ng/kg/d) of investigated AFB_1_ for females. (**D**) Cumulative probabilistic estimation of EDI (ng/kg/d) of investigated AFB_1_ for females.

The probabilistic outcomes of AFB_1_ are listed in Table 3 and Figure 1. The estimated 50th percentiles of EDI for AFB_1_ in all medical areca nut samples were 8.96 × 10^−5^ and 1.01 × 10^−4^ μg/kg/day for men and women, respectively. For the high-exposure population, the estimated 90th percentiles of EDI for medical areca nut samples were 1.53 × 10^−3^ and 1.81 × 10^−3^ μg/kg/day for men and women, respectively. The estimated 95th percentiles of EDI for medical areca nut samples were 2.74 × 10^−3^ and 3.19 × 10^−3^μg/kg/day for men and women, respectively. The maxima of EDI for AFB_1_ in medical areca nut samples were 2.71 × 10^−2^ and 2.25 × 10^−2^ μg/kg/day for men and women, respectively.

#### 2.2.2. Risk Assessment Based on the MOE Approach

Furthermore, EDI values were utilized for carcinogenic risk assessment based on both MOE and HCC approaches. Both the MOE and HCC distributions were simulated via the MCS method, one of the most recognized probabilistic models in a health risk assessment owing to its reliability and accuracy [41,42,43,44]. An MCS with 20,000 iterations was employed for both the MOE and HCC distributions to handle uncertainties in the AFB_1_ contents, exposure frequency, and ingestion rate of the medical areca nut samples. The values from these distributions were randomly selected and incorporated into the risk analysis [45,46].

**Figure 2:**
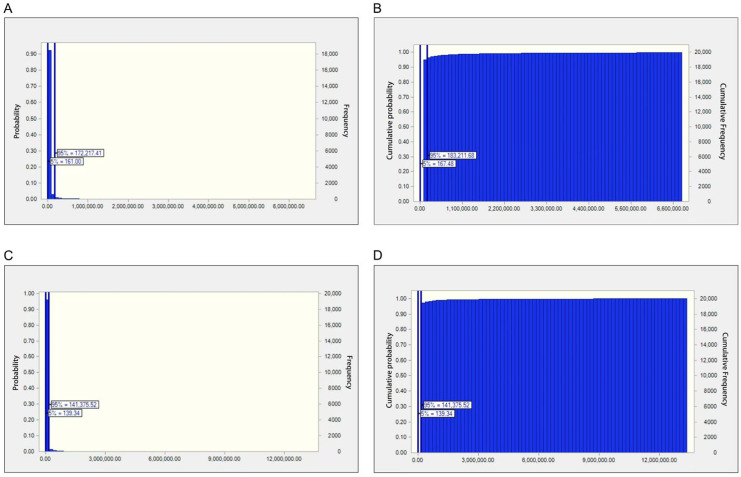
(**A**) Probabilistic estimation of MOE of investigated AFB_1_ for males. (**B**) Cumulative probabilistic estimation of MOE of investigated AFB_1_ for males. (**C**) Probabilistic estimation of MOE of investigated AFB_1_ for females. (**D**) Cumulative probabilistic estimation of MOE of investigated AFB_1_ for females.

MOEs obtained based on the BMDL_10_ for AFB_1_ in the samples are represented in Table 3 and Figure 2. The MOE values of AFB_1_ in samples from the estimated 75th percentile to the maximum were higher compared with the safe margin of 10,000 for both men and women, suggesting that the carcinogenic health risk was low. However, the MOE values of AFB_1_ in samples from the estimated 0 to 75th percentile were below the 10,000 threshold for both men and women, revealing an elevated liver cancer risk.

#### 2.2.3. Risk Assessment via the Quantitative HCC Risk Approach

**Figure 3:**
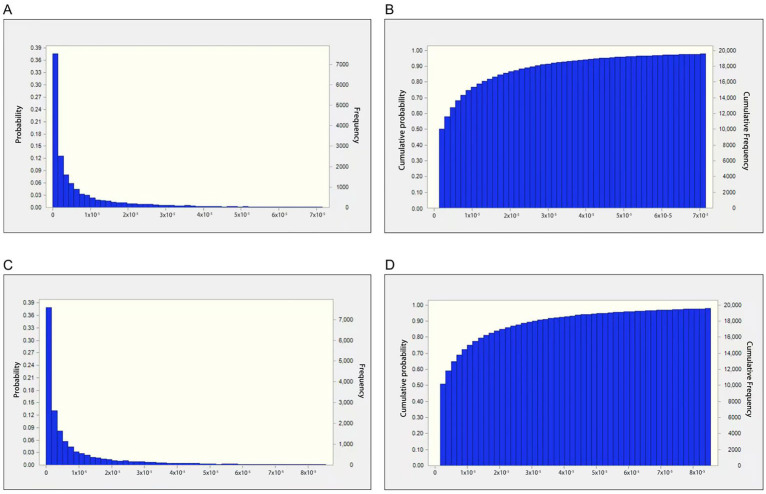
(**A**) Probabilistic estimation of HCC of investigated AFB_1_ for males. (**B**) Cumulative probabilistic estimation of HCC of investigated AFB_1_ for males. (**C**). Probabilistic estimation of HCC of investigated AFB_1_ for females. (**D**) Cumulative probabilistic estimation of HCC of investigated AFB_1_ for females.

In addition to the MOE method, AFB_1_’s risk was also characterized via the FAO/WHO-proposed quantitative HCC risk approach (Table 3 and Figure 3). The estimated minimum to 90th percentile of HCC values for males was <0.1 liver cancer cases/100,000 people, which is below 1/1,000,000 after exposure to medical areca nuts, indicating that the liver cancer risk was low. However, the estimated 90th percentile to the maximum of HCC values for men was from 0.27 to 4.04 liver cancer cases/100,000 individuals, which is higher than 1/1,000,000 upon exposure to medical areca nuts and thus of concern. The estimated minimum to 75th percentile of HCC values for females was <0.1 liver cancer cases/100,000 individuals, which is lower than 1/1,000,000 after exposure to medical areca nuts, indicating an acceptable liver cancer risk. However, the estimated 75th percentile to the maximum of HCC values for females was from 0.12 to 4.77 liver cancer cases/100,000 individuals, which is higher than 1/1,000,000 after exposure to medical areca nuts.

### 2.3. Sensitivity Analysis Results

**Figure 4:**
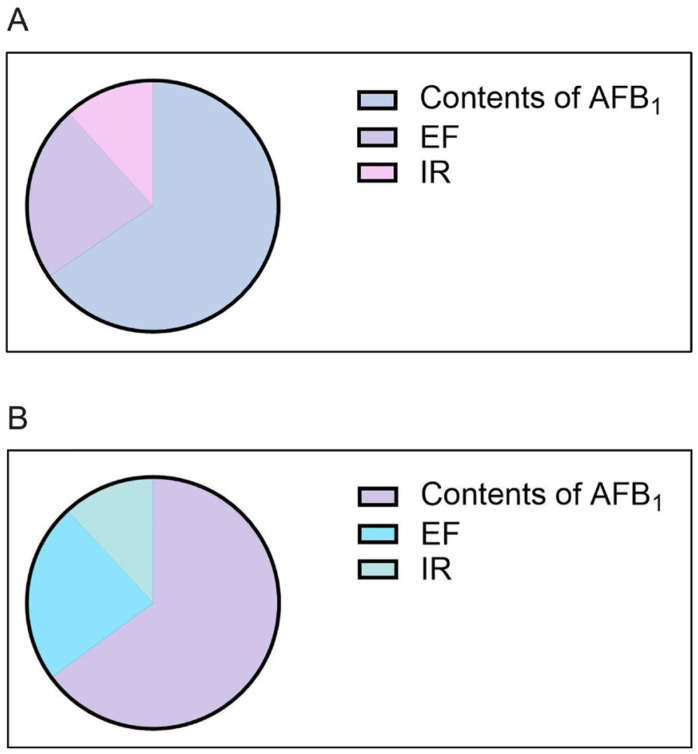
(**A**) Sensitivity analyses of concentrations of AFB1, EF, and IR for HI for males. (**B**) Sensitivity analyses of concentrations of AFB1, EF, and IR for HI for females.

Sensitivity analysis quantitatively ranks input indexes on the basis of their respective contributions to the model output’s variability and uncertainty. Figure 4 depicts sensitivity analysis data for indexes mostly affecting the estimated risk output. For both the MOE and HCC approaches, AFB_1_ content had the greatest effect on results, followed by EF and IR. The results were consistent with a recent study assessing the health risk conferred by chromium in mangrove sediments and toxicants in groundwater sources in southwestern Nigeria [47]. Therefore, reducing the levels of AFB_1_ in samples and limiting the exposure frequency and ingestion rates of medical areca nuts are considered the most beneficial suggestions for reducing the risk of cancer caused by AFB_1_ in medical areca nuts.

### 2.4. Discussion

Our previous study investigated the levels of aflatoxins in more than 220 commonly used TCM preparations, for a total of 2100 samples [48]. The overall detection rate of aflatoxins was about 7.7%, and the overall qualified rate approached 97%, on the basis of limits in the 2020 edition of the Chinese Pharmacopoeia. Even though the overall detection rate of aflatoxins in TCM is generally low, aflatoxin contamination of some types of TCM, including medical areca nuts, is of great concern. In a study by Dullah et al., 80.6% of herbal medicines had contamination with AFB_1_ at levels ranging from 0.275 to 13.941 μg/kg [49]. Another study in Thailand showed that AFB_1_ contaminations in all herbal products for various dosage forms were below the Thailand National Regulatory Limit (20 μg/kg), but some were above the European permissible limit. In addition to herbal medicines, in a study by Fang et al., aflatoxins were detected in 27.1% of the examined food samples at levels between 0.07 and 262.63 μg/kg, with AFB_1_ being the commonest in various sample types [50]. Among these samples, peanut oil showed the highest rate of aflatoxin contamination. In another study, 60 imported raw hazelnut kernels without shells had contamination with AFB_1_ at levels of 3.145 to 8.13 μg/kg [51].

Risk assessment represents an important approach for ensuring that herbal medicines are safe. It cannot be ignored that AFB_1_ contamination in TCM inherently constitutes an important threat to human health. Moreover, different age groups or populations experience different extents of risk, with variability and randomness permeating the entire process of health risk transmission, thus leading to high uncertainty and potential complexity in health risk assessment [52,53]. Uncertainty might exist in deterministic risk estimations that use single-point input factors. Therefore, how to deal with uncertainty in health risk assessments has become a new focus during risk assessments. This study used the MCS technology to quantify potential differences and complexities in the assessment process for different exposed populations. Considering the characteristics of TCM consumption, this study proposes the first comprehensive probabilistic risk assessment model using both the MOE and HCC for AFB_1_ in TCM, providing a new perspective for the health risk assessment of AFB_1_ in TCM. Probabilistic risk assessment represents an important supplement to deterministic risk assessment. The current study provides an innovative perspective for diminishing risk and supporting the rational application of TCM in the clinic.

Since the cancer risk of medical areca nut ingestion is non-negligible, especially in high-exposure individuals, implementation of risk management policies is needed to minimize risk, aiming to improve public health via the scientific utilization of medical areca nuts. Many public health strategies are employed for controlling aflatoxin exposure burden and lowering the risk of liver cancer. These interventions can be classified as cultivated, ingestion, and clinical [54]. Cultivated interventions can be applied either in the field (pre-harvest) or during storage and transportation (post-harvest) to reduce aflatoxin levels in TCM and are considered primary interventions. Ingestion and clinical interventions can be considered secondary interventions. These interventions cannot decrease actual aflatoxin contents in TCM products but can decrease the occurrence of aflatoxin-related diseases, either by declining aflatoxin’s bioavailability or by alleviating aflatoxin-induced harm. Sensitivity analysis demonstrated that AFB_1_ content was the variable with the greatest effect on probabilistic risk assessment results, followed by EF and IR. Therefore, reducing the levels of AFB_1_, further refining medical areca nut exposure frequency, and decreasing the ingestion rates are highly recommended. Moreover, because nutritional status, dietary structure, and metabolism in differently exposed people may result in uncertain risk assessment results, exploring the MCS method in combination with population-based biokinetic models is highly encouraged.

### 2.5. Conclusions

A novel comprehensive probabilistic risk assessment approach was designed to evaluate the risk of AFB_1_ exposure via medical areca nut consumption using both the MOE and liver cancer risk estimation strategies based on an MCS. Additionally, to obtain a more realistic scenario, EF values for medical areca nuts were retrieved from questionnaire data. This study is the first of this kind, demonstrating the applicability of stochastic exposure assessment tools in the precise and scientific determination of AFB_1_’s health risk in medical areca nuts, with the core purpose of minimizing cancer risk and promoting the safe usage of TCM preparations in clinical settings.

## 3. Materials and Methods

### 3.1. Sample Collection

A total of 12 provinces or municipalities were selected as the sampling sites. In total, 158 samples were collected from the Chinese TCM market, retail shops, and workshops by trained investigators from the Province of Anhui, Hebei, Sichuan, Fujian, Guangdong, Guangxi, Guizhou, Jiangsu, Jiangxi, Yunnan, Shanxi, and the City of Beijing. Samples were stored in glass containers at 4 °C until analysis within 24 h.

### 3.2. Sample Preparation and Analysis

Approximately 5 g of sample powder (sieved through a 24-mesh sieve) was obtained and precisely weighed. Then, 50 mL of a 70% methanol solution was precisely measured and added to the sample powder. The mixture was subjected to ultrasonication for 30 min and subsequently centrifuged (4000 r/min for 10 min). A total of 10 mL of the resultant supernatant was precisely measured and diluted to 20 mL with water, and then shaken well. Next, 3 mL of the diluted supernatant was precisely measured and slowly passed through a pre-treated Hydrophile-Lipophile Balance (HLB) column [3 mL (60 mg), eluted sequentially with 3 mL each of methanol and water], allowing an appropriate amount of air to pass through, after which the eluent was collected. Subsequent elution was performed with 3 mL of methanol, and the eluent was collected. Both eluent samples were combined and dried slowly with nitrogen gas at 40 °C until near-dryness. The sample volume was adjusted to 1 mL with a 50% acetonitrile solution, followed by filtration using a 0.22 μm microporous filtration membrane. The resulting filtrate was employed for further testing.

### 3.3. Analytical Procedure

An 8060-LC-MS/MS coupled with an electrospray ionization (ESI) source (Shimadzu, Kyoto, Japan) was utilized for measurements. Chromatographic separation of AFB_1_ employed a Waters ACQUITY BEH C18 column (50 mm × 2.1 mm, 1.7 μm, Waters, Milford, MA, USA), with the column temperature at 30 °C and flow rate at 0.3 mL/min. Gradient elution was conducted with water (with 0.01% formic acid) and acetonitrile/methanol (1:1, *v*/*v*) in channels A and B, respectively, as follows: 0 min, 65% A; 4.5 min, 15% A; 6 min, 0% A; 6.5 min, 65% A; and 10 min, 65% A. ESI was utilized as the ion source, and the positive ion mode was adopted. Collision energies (CEs) were 50 V. The electrospray voltage was 2500 V, the ion source temperature was 150 °C, and the N_2_ flow rate was 650 L/h. The sample injection volume was 5 μL. For analytical quality assurance, chemical blanks, spikes, and triplicates were analyzed throughout the analytical process. Each sample was run in triplicate, and the final values were an average of the three values. The detection limit of the method was calculated based on a signal-to-noise ratio of 10 (10 S/N), which was 1 µg/kg. The accuracy of the method was controlled by the mean recovery rates of the samples (Table S1).

### 3.4. Exposure Assessment

In this study, to assess AFB_1_’s cancer risk, a real-life exposure scenario was simulated via the calculation of EDI (μg/kg bw/day) [55,56]:

EDI = EF × Ed × IR × C/W × AT(1)

where EDI represents the estimated daily intake of AFB_1_ in medical areca nuts; EF is the exposure frequency; Ed represents the exposure time, i.e., 20 years; IR is the daily intake rate of medical areca nuts (g/day). C represents AFB_1_ amounts in medical areca nuts (μg/g); W is the mean body weight, i.e., 69.6 and 59 kg for male and female participants, respectively; AT represents the mean duration of the exposure to medical areca nuts, i.e., 365 days/year × 70 years.

### 3.5. Risk Characterization

To assess health risks, two modeling approaches proposed by international regulatory agencies were applied, involving the MOE strategy approach suggested by the EFSA [40] and a quantitative hepatocellular carcinoma (HCC) risk strategy developed by the FAO/WHO [57].

#### 3.5.1. The MOE Approach

The EFSA Scientific Panel on Contaminants in the Food Chain [48] suggested the utilization of the benchmark dose lower confidence limit for 10% extra risk (BMDL_10_) for MOE characterization. To assess AFB_1_ risk, MOE values for AFB_1_ were determined as the BMDL_10_ of 250 ng/kg bw/day for AFB_1_ by EDI [49]. In general, an MOE value < 10,000 indicates an alarming cancer risk for humans. The value of 10,000 encompasses an index of 100 for intraspecies and interspecies differences, a factor of 10 to account for the BMDL_10_ linking to a measurable 10% tumor incidence, and an additional factor of 10 for human variability. It should be clarified that MOE values do not quantify the risk but only reveal a level of concern, which suggests that the higher the MOE value, the lower the level of concern.

#### 3.5.2. Quantitative HCC Risk Approach

The FAO/WHO proposed a method to quantitate the risk of liver cancer caused by AFB_1_ exposure, considering AFB_1_’s carcinogenic ability [57]. In this method, considering the synergistic liver cancer effects of HBV infection, and AFB_1_ representing the core strategy for calculating AFB_1_’s carcinogenic potency (P_cancer_) [57], the P_cancer_ of AFB_1_ is determined by utilizing the HBV infection rate in a certain population, which takes into consideration both the P_cancer_ for individuals suffering from HBV infection (HBsAg+) and those without HVB infection (HBsAg−).

P_cancer_ = 0.01 × %HBsAg− + 0.3 × %HBsAg+ (2)

HCC = EDI × P_cancer_ (3)

The HBsAg+ value for Chinese individuals in this study was considered to be 7%, which was reported by Dr. Zhuang from Peking University at the 14th International Conference on Viral Hepatitis and Liver Disease. It has been calculated that the P_cancer_ value of AFB_1_ in China is 0.03. Generally, an HCC value ≤ 10^−6^ indicates carcinogenic effects have low odds of occurrence [56]. In line with the above, in our study, 0.1 cancer cases/100,000 people was considered an appropriate value for cancer risk assessment. The latter criterion reflects 1 additional cancer case in 1 million people following exposure to medical areca nuts.

#### 3.5.3. Probabilistic Assessment via Monte Carlo Simulation (MCS)

Further, a probabilistic assessment by the MCS approach was applied to minimize the overall uncertainty of input variables. An MCS with 20,000 iterations was employed by Crystal Ball (version 11.1.1.1, Oracle, Inc., Austin, TX, USA) to handle uncertainties in the AFB_1_ content, exposure frequency, and ingestion rate of medical areca nuts. Next, the probabilistic distributions of the desired input variables were investigated. Values from these distributions were randomly selected and incorporated into the risk analysis.

Here, face-to-face questionnaire data were utilized for the development of EF’s probability distribution. The probability distribution of the IR of medical areca nuts was determined, according to the Chinese Pharmacopoeia, as 3 to 10 g. AFB_1_ concentrations were also simulated with the best probability distribution applied to exposure assessment equations.

### 3.6. Data Analysis

Statistical analysis was performed using SPSS 22.0 (IBM Corporation, New York, NY, USA). MCS with 20,000 iterations was employed with Crystal Ball (version 11.1.1.1) to handle uncertainties in the AFB_1_ content, exposure frequency, and ingestion rate of the samples. Figures were plotted using the GraphPad 9.0 software (GraphPad, San Diego, CA, USA).

## Figures and Tables

**Table 1:** Contents of AFB_1_ in all the medical areca nut samples and stir-fried areca nut samples (µg/kg).

	Min	P25	Mean	P50	P75	Max
Medical areca nut	Non-detected	1	7.7	3	18	146
Stir-fried medical areca nut	Non-detected	Non-detected	13.0	1.5	12.5	31

**Table 2:** The optimum probability distribution of the parameters simulated in the Monte Carlo simulation technique.

Parameter	EF	IR	Contents of AFB_1_
Type of distribution	Triangular distribution	Uniform distribution	Weibull distribution

EF: exposure frequency; IR: ingestion rates.

**Table 3:** Probabilistic estimation of EDI (ng/kg/d) and MOE of investigated AFB_1_.

		P_5_	P_10_	P_25_	P_50_	P_75_	P_90_	P_95_	Max
Male	EDI	1.51 × 10^−3^	4.35 × 10^−3^	2.07 × 10^−2^	9.24 × 10^−2^	0.33	0.89	1.50	13.48
	MOE	166.40	280.96	756.01	2704.11	12,077.28	57,475.22	165,247.18	401,863,050.72
	HCC	4.53 × 10^−4^	0.001	0.006	0.028	0.099	0.267	0.450	4.044
Female	EDI	1.78 × 10^−3^	5.13 × 10^−3^	2.44 × 10^−2^	0.11	0.39	1.05	1.77	15.90
	MOE	139.96	238.25	619.16	2320.56	10,659.60	50,906.95	148,286.36	517,480,061.53
	HCC	5.35 × 10^−4^	0.002	0.007	0.033	0.117	0.315	0.531	4.770

EDI: estimated daily intake; MOE: the margin of exposure; HCC: liver cancer risk.

## Data Availability

The original contributions presented in this study are included in the article/Supplementary Materials. Further inquiries can be directed to the corresponding authors.

## Supplementary Materials

The following supporting information can be downloaded at https://www.mdpi.com/article/10.3390/toxins17050252/s1, Table S1: Results of recovery rates of AFB1 (%).
