# Supplementary material for: A Comprehensive Probabilistic Risk Assessment Strategy of Aflatoxin B1 Exposure from Medical Areca Nuts Consumption
Source: Toxins (Basel). 2025 May 19;17(5):252. doi: 10.3390/toxins17050252 (PMC12115991; doi:10.3390/toxins17050252)
Supplement: Supplementary file 1 [file toxins-17-00252-s001.zip › toxins-3557390-supplementary.pdf]

Supplementary Materials: A Comprehensive Probabilistic Risk Assessment Strategy of Aflatoxin B1 Exposure from Medical Areca Nuts Consumption

Table S1. Results of recovery rates of AFB_1(%).

Added amounts	2μg/kg	10μg/kg	50μg/kg
Recovery rates	82±3.6	92±2.3	89±2.3
